# Long-term cerebral white and gray matter changes after preeclampsia

**DOI:** 10.1212/WNL.0000000000003765

**Published:** 2017-03-28

**Authors:** Timo Siepmann, Henry Boardman, Amy Bilderbeck, Ludovica Griffanti, Yvonne Kenworthy, Charlotte Zwager, David McKean, Jane Francis, Stefan Neubauer, Grace Z. Yu, Adam J. Lewandowski, Yrsa Bergmann Sverrisdottir, Paul Leeson

**Affiliations:** From the Radcliffe Department of Medicine (T.S., H.B., Y.K., C.Z., J.F., S.N., A.J.L., P.L.), Department of Psychiatry (A.B.), Nuffield Department of Clinical Neurosciences (L.G.), Nuffield Department of Surgical Sciences (Y.B.S.), and Department of Cardiology (G.Z.Y.), University of Oxford; Department of Radiology (D.M.), Stoke Mandeville Hospital, Aylesbury, UK; and Department of Neurology (T.S.), University Hospital Carl Gustav Carus, Technische Universität Dresden, Dresden, Germany.

## Abstract

**Objective:**

To determine whether changes in cerebral structure are present after preeclampsia that may explain increased cerebrovascular risk in these women.

**Methods:**

We conducted a case control study in women between 5 and 15 years after either a preeclamptic or normotensive pregnancy. Brain MRI was performed. Analysis of white matter structure was undertaken using voxel-based segmentation of fluid-attenuation inversion recovery sequences to assess white matter lesion volume and diffusion tensor imaging to measure microstructural integrity. Voxel-based analysis of gray matter volumes was performed with adjustment for skull size.

**Results:**

Thirty-four previously preeclamptic women (aged 42.8 ± 5.1 years) and 49 controls were included. Previously preeclamptic women had reduced cortical gray matter volume (523.2 ± 30.1 vs 544.4 ± 44.7 mL, *p* < 0.05) and, although both groups displayed white matter lesions, changes were more extensive in previously preeclamptic women. They displayed increased temporal lobe white matter disease (lesion volume: 23.2 ± 24.9 vs 10.9 ± 15.0 μL, *p* < 0.05) and altered microstructural integrity (radial diffusivity: 538 ± 19 vs 526 ± 18 × 10^−6^ mm^2^/s, *p* < 0.01), which also extended to occipital and parietal lobes. The degree of temporal lobe white matter change in previously preeclamptic women was independent of their current cardiovascular risk profile (*p* < 0.05) and increased with time from index pregnancy (*p* < 0.05).

**Conclusion:**

A history of preeclampsia is associated with temporal lobe white matter changes and reduced cortical volume in young women, which is out of proportion to their classic cardiovascular risk profile. The severity of changes is proportional to time since pregnancy, which would be consistent with continued accumulation of damage after pregnancy.

Women with a history of preeclampsia have a 2-fold higher risk of cerebrovascular disease.^1^ This may be because women who had preeclampsia also have a higher cardiovascular risk burden in later life. Alternatively, it has been proposed that structural and functional changes to the vasculature at the time of preeclamptic pregnancy may contribute to increased cerebrovascular risk.

Indeed, preeclampsia is associated with development of generalized endothelial dysfunction shown to be related to several factors, including placental disease and maternal inflammatory stimuli during pregnancy.^2^ This endothelial dysfunction has been shown to be present for several years after preeclampsia and, in addition, a persistent state of enhanced response to vascular injury has been demonstrated that results in increased vascular smooth muscle cell proliferation and vessel fibrosis.^3^ This could result in an increased cerebrovascular vulnerability to cardiovascular risk factors that extends beyond pregnancy.^4^

Cerebral white matter changes can be quantified as hyperintense lesions on MRI to generate an imaging-derived phenotype of brain damage in younger people. Earlier, more subtle changes in white matter integrity that precede white matter lesions (WMLs) can also now be identified with diffusion tensor imaging (DTI) indices of microstructural integrity.^5,6^ These white matter changes are known to predict risk of future stroke, dementia, and death, which is also associated with impaired cerebral perfusion as characterized by degree of gray matter atrophy.^7,8^

We used these imaging-derived measures to confirm previous reports of increased cerebral damage long term after preeclampsia and then studied in detail the pattern of brain damage in these young women.^9–12^ We then studied whether the changes related to pregnancy history or merely reflected the current cardiovascular risk profile. Finally, we assessed whether there was evidence of more rapid accumulation of damage in women with a history of preeclampsia.

## METHODS

### Participants and protocol.

We identified women who gave birth in the John Radcliffe Hospital between 5 and 15 years prior to the study with a discharge diagnosis of preeclampsia. We confirmed diagnosis of preeclampsia based on standard International Society for the Study of Hypertension in Pregnancy definitions.^13^ In parallel, we identified women who had a confirmed normotensive pregnancy in the same years, without history of hypertensive pregnancy in previous or subsequent pregnancies, as controls. Screening procedures, exclusion criteria, and cardiovascular risk assessment are detailed in appendices e-1 and e-2 at Neurology.org. In this case control study, participants underwent MRI with a 1.5T scanner (Magnetom Avanto, Siemens, Munich, Germany) and 12 element head matrix coil.

### Standard protocol approvals, registrations, and patient consents.

The study was approved by the Oxfordshire Ethics Committee A (ethics reference number 08/H0604/127). Written informed consent was obtained from all participants.

### Brain volumes: Total, white matter, and gray matter.

Total brain and white and gray matter volumes were assessed in T1-weighted sequences. Gray matter volumes normalized to skull size were quantified in a fully automated fashion using the SIENAX algorithm with adjustment for skull size as previously described.^14^ Details are provided in appendix e-3. The model-based segmentation/registration tool FSL FIRST was applied to analyze volumes of subcortical structures (thalamus, caudate, putamen, pallidus, hippocampus, amygdala, accumbens, brainstem gray matter).^15^

### White matter: Number and volume of lesions.

**Figure 1:**
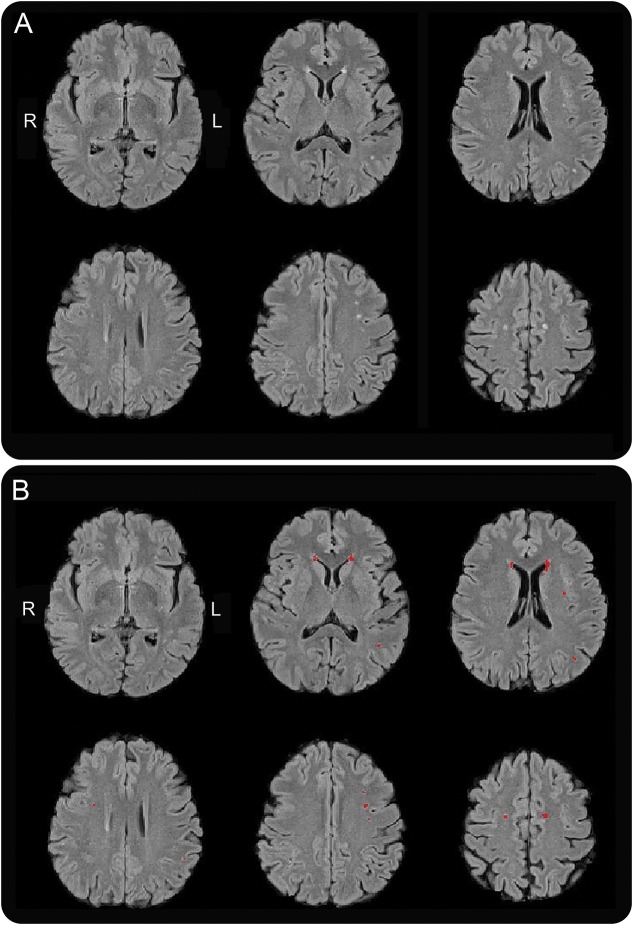
Voxel-based detection of white matter lesions on fluid-attenuated inversion recovery (FLAIR) MRI Voxel-based segmentation in a woman with a history of preeclampsia with burden of lesions that is representative of our cohort. Transversal T2 FLAIR images (A) before and (B) after voxel-based segmentation using voxel-based segmentation software. Detected lesions are highlighted in red.

All WML analyses were performed in the total brain and the temporal, frontal, parietal, and occipital lobes. T2-weighted, fluid-attenuated inversion recovery (FLAIR) sequences were analyzed visually by 2 experienced operators blinded to the experimental groups (D.M., T.S.) separately for number of WML. Fazekas scale was calculated to assess severity of WML.^16^ Numbers and volumes of WML were also automatically segmented on FLAIR images with a newly developed tool, Brain Intensity Abnormality Classification Algorithm (BIANCA), a fully automated, supervised method for WML detection, based on the k-nearest neighbor algorithm to create a probability map of WML (figure 1).^17^ Details are provided in appendix e-4.

### White matter: Microstructural integrity.

DTI was used to assess white matter microstructural integrity. Image analysis using tract-based spatial statistics was performed, as previously described.^18^ Mean diffusivity, fractional anisotropy, radial diffusivity, and axial diffusivity were calculated for the whole white matter as well as for the temporal, frontal, parietal, and occipital lobe. Details are provided in appendix e-5.

### Statistical analysis.

Statistical analysis was performed using SPSS version 20 (IBM, Armonk, NY). We performed a complete case analysis. In text and tables, continuous data are presented as mean (±SD). WML count is reported as median (range) as the data were all distributed nonparametrically. In bar graphs, error bars indicate standard error of the mean. Group differences between women with previous preeclampsia and controls were analyzed using Student *t* test or Mann-Whitney *U* test, according to distribution. Dichotomous data were compared using χ^2^ test. The α level for statistical significance was set to 0.05. Univariate linear regression models were built to assess how cardiovascular risk factors and pregnancy-related factors associated with brain structural integrity. Unstandardized β coefficients (β) and *p* values were calculated. Multivariate regression models were built to assess the association of history of preeclampsia and brain structure with adjustment for cardiovascular risk factors. All factors that emerged as predictor variables in the univariate analysis were included in a stepwise regression model as candidate variables and then removed by stepwise backward selection procedure with removal threshold set at *p* = 0.2 to identify the strongest independent predictors of structural brain changes. Pearson or Spearman correlation analyses were performed where appropriate to assess associations between white matter microstructure and time from index pregnancy separately in previously preeclamptic women and controls.

## RESULTS

### Participants.

Images from 87 women were studied, 4 of which were excluded from the analyses because MRI revealed patterns of lesions consistent with chronic inflammatory brain disease. Therefore, 34 women with a history of preeclampsia and 49 normotensive pregnancy control women were included. There were no missing data. Characteristics of the study group are detailed in table 1 and appendix e-6 and show, as expected, women with a history of preeclampsia have higher blood pressures 5–15 years after pregnancy and, of these women, 3 were on antihypertensive medication.

**Table 1:**
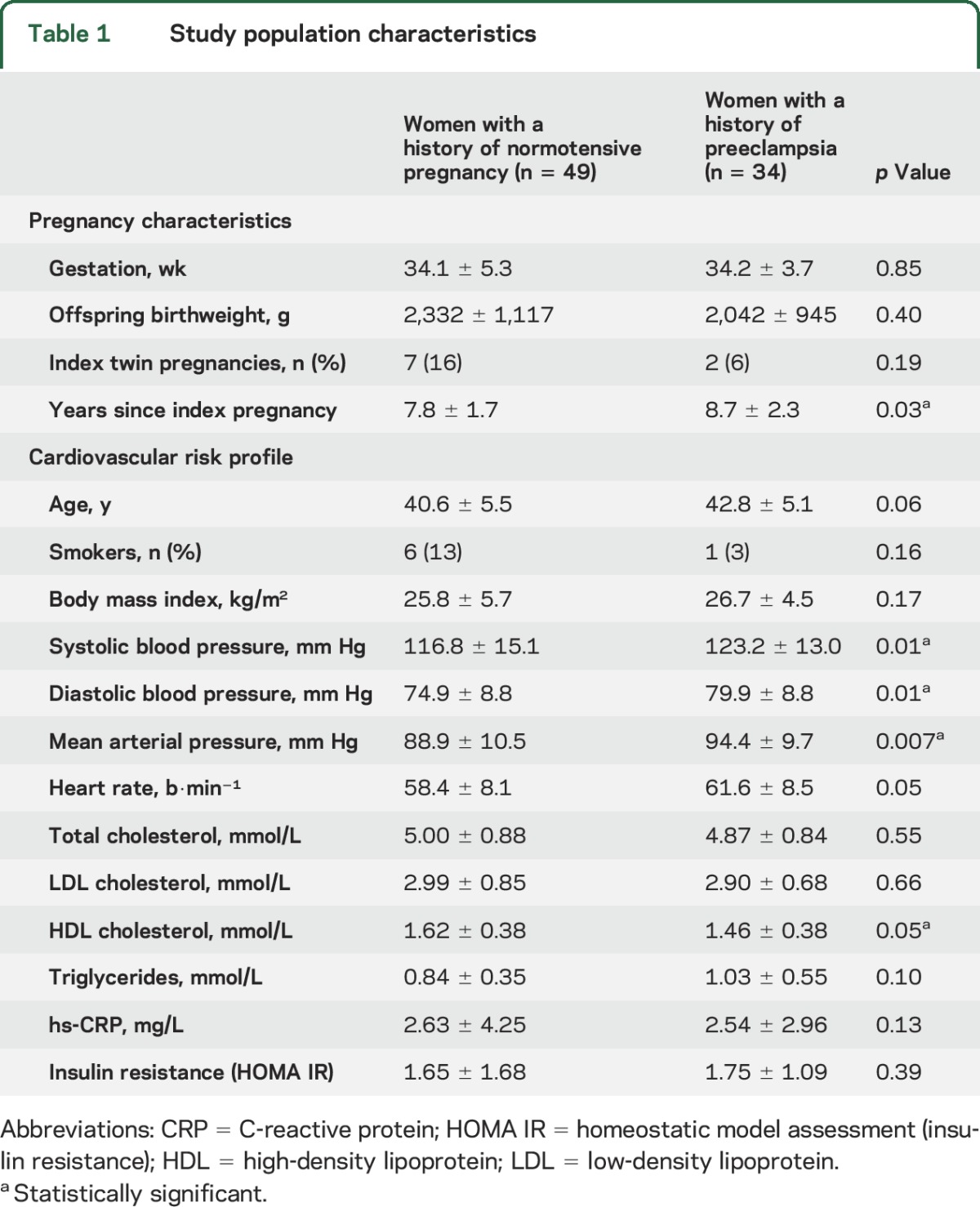
Study population characteristics

### Gray matter and brain volumes.

**Figure 2:**
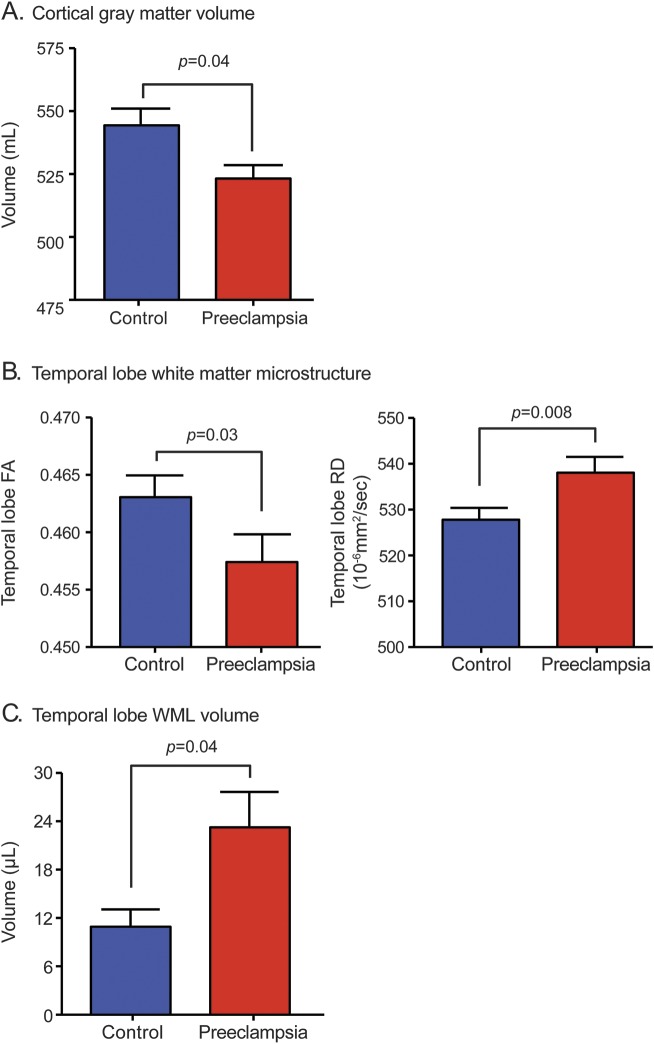
Structural brain changes in the temporal lobe The bar graphs illustrate impaired white matter structural integrity in the temporal lobe as well as gray matter in the analysis of the total brain in women with a history of preeclampsia (red bars) compared to those who had normotensive pregnancy (blue bars). Among computed measures of brain structure, (A) volumetric assessment of lesions shows greater damage to the temporal lobe white matter, (B) evaluation of white matter microstructure via diffusion tensor imaging analyzed for fractional anisotropy (FA) and radial diffusivity (RD) demonstrates impairment, and (C) volumetric assessment of gray matter structure shows reduced cortical volume. WML = white matter lesion.

Cortical gray matter volume was reduced in women with a history of preeclampsia compared with normotensive pregnancy control women (figure 2A). This was also true when total gray matter (also including subcortical structures) was compared between groups (661.2 ± 142.6 mL preeclampsia vs 686 ± 54.2 mL normotensive pregnancy, *p* = 0.04). However, there were no differences in volumes of individual subcortical gray matter structures (table e-1), total brain volume (1,600.3 ± 83 mL preeclampsia vs 1,621 ± 71 mL normotensive pregnancy, *p* = 0.23), or white matter volume (905 ± 55 mL preeclampsia vs 901 ± 58 mL normotensive pregnancy, *p* = 0.76).

### White matter structural integrity.

Both groups of women had substantial amounts of frontal lobe white matter damage and, as a result, there was no significant difference in total number of WML based on visual analysis, voxel-based automated measures (table e-2), or Fazekas scores (1.6 ± 0.9 vs 1.4 ± 0.8, *p* = 0.29). However, regional voxel-based analysis revealed a significant increase in temporal lobe WML volume in preeclamptic women when compared to those who had normotensive pregnancy (figure 2A). Temporal lobe differences were also evident on the diffusion tensor microstructural imaging, which revealed lower fractional anisotropy and higher radial diffusivity (figure 2B) in women with a history of preeclampsia compared to normotensive pregnancy control women. Furthermore, although WML volume was not higher in the parietal and occipital lobe, there was evidence of changes at the microstructural level in these regions with higher radial diffusivity as well as lower occipital lobe fractional anisotropy (table e-3).

### Cardiovascular risk factors or preeclampsia as predictors of cerebral structural damage.

In the bivariate regression analyses, blood pressure and serum concentration of high-density lipoprotein (HDL) cholesterol showed a significant association with temporal white matter changes (table 2). However, while blood pressure was linked to both microstructural white matter changes and WML volume, HDL cholesterol was related only to microstructural damage. To further elucidate whether preeclampsia is independently associated with structural changes or rather reflects accumulative burden of cardiovascular risk factors, multivariate models were built. These models demonstrated associations of history of preeclampsia with both WML volume (β = 0.01, *p* = 0.003) and microstructural impairment assessed via radial diffusivity (β < 0.01, *p* = 0.049) that were independent of age and blood pressure (β = 0.01, *p* = 0.003).

**Table 2:**
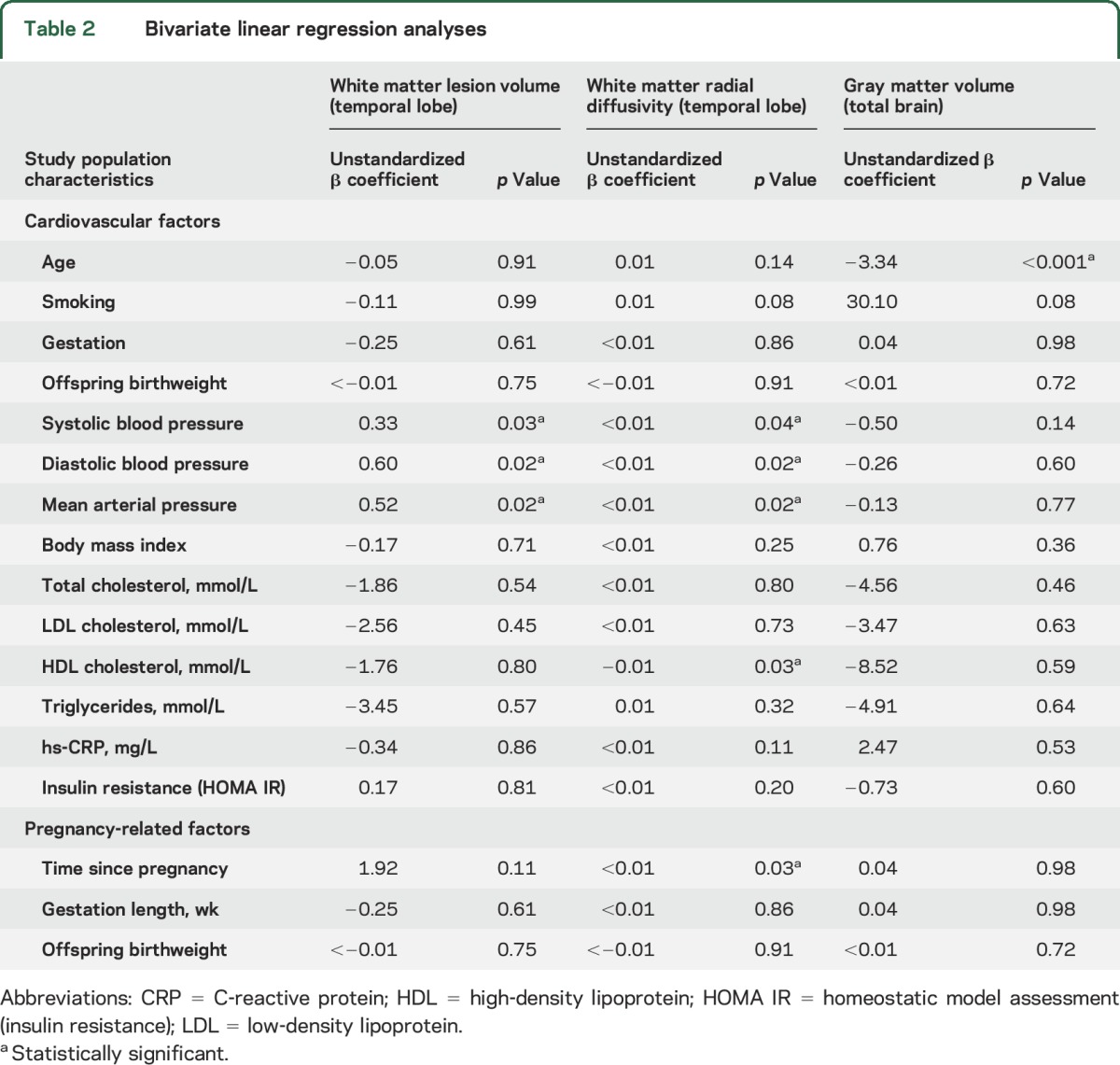
Bivariate linear regression analyses

### Fixed or cumulative changes in cerebrovascular structure after preeclampsia.

**Figure 3:**
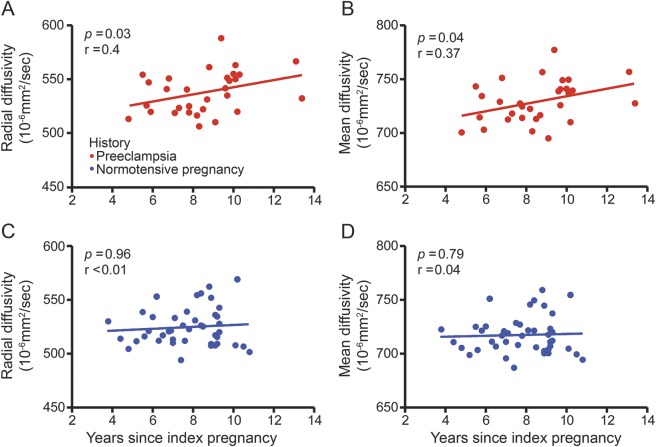
Correlation of temporal white matter microstructure and time since pregnancy The scatterplots show that time since index pregnancy was positively correlated with white matter microstructural changes assessed via (A) radial and (B) mean diffusivity in women with a history of preeclampsia. By contrast, in women who had normotensive pregnancy, neither (C) radial diffusivity nor (D) mean diffusivity was correlated with time since index pregnancy.

We were not able to study whether there was cumulated brain structural changes due to repeated episodes of preeclampsia as only 5 women in our study population had more than 1 hypertensive pregnancy. However, we investigated whether there was a fixed effect of pregnancy or whether the association between preeclampsia and degree of structural impairment changed over time. Gray matter and temporal white matter lesion volume did not vary with severity of preeclampsia defined as early or late onset (appendix e-7 and table e-4) but, as illustrated in figure 3, there was a significant correlation between white matter microstructural impairment and time from index pregnancy in previously preeclamptic women as well as with volumetric WML assessment (table 2). Interestingly, this association was not evident in those who had normotensive pregnancies, who had a constant degree of cerebrovascular change over time. In stepwise regression models that included all variables that were associated with brain damage in univariate analysis, history of preeclampsia (β = 1.5, *p* = 0.009) and time from index pregnancy (β = 3.1, *p* = 0.03) emerged as predictors of temporal white matter microstructural impairment (radial diffusivity) while temporal lesion volume was predicted only by history of preeclampsia (β = 12.7, *p* = 0.01). For cortical volume loss, age emerged as the main predictor (table 2).

## DISCUSSION

This study shows that women 5–15 years after preeclampsia have greater white matter structural changes in the temporal lobes and lower cortical gray matter volume than women who have normotensive pregnancies. Increased temporal lobe burden is not explained by their higher cardiovascular risk profile. Furthermore, in contrast to women who have normotensive pregnancies, the degree of structural impairment increases with time, consistent with a persistent susceptibility after pregnancy.

Long-term differences in cerebral structure have previously been noted in women who had preeclampsia. In a subpopulation of the Family Blood Pressure Project Genetic Epidemiology Network of Arteriopathy (GENOA) study aged 58 ± 10 years, total brain volumes were reduced in those who had preeclampsia.^12^ Our cohort was approximately 20 years younger at the time of assessment and, at this stage, we observed lower volumes in the cortical gray matter but not total brain. Our finding of reduced total gray matter with similar subcortical volumes supports a specific reduction in cortical volumes, while the similar total brain volume between groups may relate to some enlargement of CSF spaces in compensation for the cortical atrophy or merely relate to the power of our study to identify relatively small proportional differences in total brain volume. However, we found degree of cortical volume loss was significantly related to age, and together these studies might suggest an age-dependent decline of cortical volume beyond the time of pregnancy that eventually leads to reduction in total brain volume. Our finding of significantly lower cortical volume after preeclampsia warrants follow-up research to study the characteristics of post preeclamptic cortical changes longitudinally, how these relate to functional cerebral impairment, and whether these might be modified by intensified management of cardiovascular risk factors.

The GENOA subpopulation has also been analyzed for WML burden in total brain, which was increased in women with a history of preeclampsia, but interpretability of this observation was limited by the lack of regional WML assessment.^12^ By contrast, we found that total brain lesions were similar across the cohort due to a large number of frontal lobe lesions in both groups. However, those who had had preeclampsia had more extensive changes that involved the temporal, as well as occipital and parietal, lobes. Furthermore, the degree of temporal lobe white matter damage relates to time since index pregnancy in those with previous preeclampsia but does not show such an association in women who had normotensive pregnancies. If temporal lobe burden continues to increase after preeclampsia, the more advanced age of the GENOA subpopulation might explain why differences in total WML become evident by age 60 years. Alternatively, the differences may reflect our methodologic approach to quantification of WML. Compared to another study of total brain WML volume changes in the total brain after preeclampsia that reported higher levels, we had a thinner slice thickness and did not exclude WML based on presentation on T1-weighted images, since variability of T1 signal intensity of pathologic WML ranging from hypointensity to hyperintensity is well-reported.^19,20^ Although our approach is more consistent with clinically validated characterization of WML, it does result in identification of smaller WML and overall higher volumetric scores. This might reduce variability between groups and make it more difficult to identify differences at a whole brain level. Nevertheless, our findings of increased frontal lobe damage in both groups are consistent with a previous MRI study that applied a single rater-based manual evaluation of WML.^11^ In contrast to our data, this regional white matter analysis showed no differences of temporal white matter changes between previously preeclamptic women and normotensive pregnancy controls. However, this observation may have been limited by rater-dependent variability due to the visual approach of analyzing brain scans without automated voxel-based segmentation and by the absence of assessment of white matter microstructure.

A major strength of our study has been the ability to undertake both regional and microstructural assessment. This has highlighted that although the frontal lobe has the most pronounced number of WML, involvement of other brain regions varies considerably with pregnancy history. Interestingly, a previous study in 77 adults using DTI indicated that age-related microstructural damage is most pronounced in the anterior brain regions but evolves posteriorly. In this study, the presence of arterial hypertension led to emergence of lesions in the temporal and occipital lobe, similar to the patterns we observed.^21^ A predominance of white matter microstructural damage related to age and hypertension in anterior brain regions was also observed in an analysis of the third generation of the Framingham Heart Study, which included young individuals at similar ages to our cohort.^6^ These studies did not evaluate the effects of preeclampsia on brain architecture but our analysis would suggest this risk factor, independent of blood pressure and age, leads to similar patterns of progressive white matter changes from anterior to posterior regions.

The mechanism for these white matter changes in relation to preeclampsia remains unclear but may relate to the pregnancy itself. Preeclampsia is considered to be triggered by placental dysfunction, which results in widespread endothelial dysregulation with consecutive deficits in perfusion of several organs, including the brain.^22^ Animal models of placental ischemia have demonstrated impaired cerebral blood flow autoregulation and increased blood–brain barrier permeability.^23^ Interestingly, the cerebral insult may not be limited to the time of pregnancy; a mouse model of preeclampsia recently showed that after preeclamptic but not normotensive pregnancy carotid injury leads to enhanced vascular remodeling with increased vessel fibrosis.^2^ This offers a plausible explanation for our finding that the amount of cerebral damage increases with time from pregnancy only in those women who had preeclampsia. This could also be a major factor underlying the increased risk of cerebrovascular disease in women with a history of preeclampsia.^1^

Another possible link between altered brain architecture beyond aging and hypertension-related changes might be autonomic dysregulation of cerebrovascular hemodynamics with impaired sympathetic modulation of cerebrovascular resistance.^24^ Higher sympathetic nerve activity after preeclampsia is more common even 40 years after pregnancy in those who are still hypertensive.^25,26^ Moreover, decreased baroreflex sensitivity and reduced heart rate variability were reported in preeclamptic women, indicating complex disturbances in autonomic regulation that might exceed pure sympathetic overactivity and contribute to sustained adverse blood pressure characteristics and cerebrovascular disease in later life.^27–29^ Our finding of greater damage to the temporal lobe in women after preeclampsia might be of functional relevance as the temporal lobe harbors centers and pathways of autonomic cardiovascular control.^30,31^

A limitation of our study is the absence of cognitive, autonomic, and cerebrovascular function assessment, which might have helped elucidate whether and by what mechanism observed brain damage translates into cerebrovascular risk and cognitive impairment.^22,24^ However, our data form a basis for follow-up research to assess these potential associations and identify targets for preventive treatment. Also, individuals with diabetes or cardiovascular disease were excluded, and therefore our results may underestimate structural changes found in the general population. Generalizability of our study may be limited due to its single-center design. A strength of our study is the comprehensive brain structural analysis enabled by a combination of advanced white and gray matter analysis techniques in a well-characterized cohort of previously preeclamptic women.

The presence of brain damage beyond a degree that can be explained by cardiovascular risk factors, which increases with time, implies that 2 clinically relevant interventions may be of value. First, there may be value in more aggressive cardiovascular prevention strategies after preeclampsia to reduce cumulative and ongoing damage. This may include targeted pharmacologic and lifestyle interventions.^32^ Second, more careful attention to neurologic symptoms may be needed in case the differences in cortical volumes and white matter changes are harbingers of an increased risk of cognitive complaints including disturbances of memory and concentration.^33–35^ Whether these cognitive disturbances relate to an increased risk of dementia is unknown but a recent survey-based study did not find higher prevalence of history of hypertensive pregnancies among women with Alzheimer disease compared to women without Alzheimer disease.^36^ It is, however, noteworthy this analysis excluded vascular forms of dementia.

Taken together, we report greater changes in temporal white matter and lower cortical gray matter volume in women with a history of preeclampsia compared with women who had normotensive pregnancies. The degree of changes in the brain is predicted by the time from affected pregnancy, which would be consistent with continued damage postpregnancy. Clinical management at the time of pregnancy and ongoing risk factor modification after delivery are both likely to be important in reducing cerebrovascular disease in later life.

## Supplementary Material

Data Supplement

Accompanying Editorial

## GLOSSARY

DTI: diffusion tensor imaging
FLAIR: fluid-attenuated inversion recovery
GENOA: Genetic Epidemiology Network of Arteriopathy
HDL: high-density lipoprotein
WML: white matter lesion
