# Supplementary material for: Long-term cerebral white and gray matter changes after preeclampsia
Source: Neurology. 2017 Mar 28;88(13):1256–64. doi: 10.1212/WNL.0000000000003765 (PMC5373775; doi:10.1212/WNL.0000000000003765)
Supplement: Data Supplement [file supp_88_13_1256__index.html]

Long-term cerebral white and gray matter changes after preeclampsia — Data Supplement 

# Long-term cerebral white and gray matter changes after preeclampsia

## Data Supplement

**Neurology® data supplements are not copyedited before publication. Published editorials and translations have been copyedited.  
 © 2017 American Academy of Neurology.  
  
 Files in this Data Supplement:**

- Appendicies - PDF
- Table e-1 - PDF
