# Supplementary material for: Long-term cerebral white and gray matter changes after preeclampsia
Source: Neurology. 2017 Mar 28;88(13):1256–64. doi: 10.1212/WNL.0000000000003765 (PMC5373775; doi:10.1212/WNL.0000000000003765)
Supplement: Data Supplement [file supp_WNL.0000000000003765_Appendices.pdf]

Supplemental data: appendices

Appendix e-1. Screening procedures

Potential study participants (both women with a history of preeclampsia and women with a history of normotensive pregnancy) were identified from the John Radcliffe Hospital Maternity records and were contacted starting with those identified from the 1998 records. All women who interacted with the maternity services were included in the clinical database with data incorporated on complications of pregnancy such as preeclampsia. Study visits for brain magnetic resonance imaging were performed between 2013 and 2015 as part of a subgroup study of a larger cohort. Criteria of preeclampsia included new onset hypertension after 20 weeks gestation in index pregnancy, diastolic blood pressure > 90mmHg on two separate occasions within a 24 hour period, new onset proteinuria of 300mg/24h or more of protein in a 24 hour urine collection or >30mg of protein/mmol of creatinine in a single urine sample or at least 2+ protein at least twice on consecutive dipstick testing. Exclusion criteria were serious mental illness at any time, pre-existing malignancy, diabetes, renal, cardiac, or vascular disease, current pregnancy, pregnancy in the past six months. By design, control subjects were recruited with a history of uncomplicated pregnancy as well as those with a history of normotensive pregnancy complicated by preterm delivery. This was to achieve frequency matching of gestation between control and preeclampsia groups and allow analysis of the effect of hypertension during pregnancy independent from preterm delivery.

Appendix e-2. Cardiovascular risk assessment

Participants were invited to attend a study visit on a single day after having fasted for at least eight hours. Medical history including cardiovascular risk factor profile and obstetric history were obtained. Participants were evaluated for markers of cardiovascular risk; including blood pressure, body mass index, insulin resistance and serum concentrations of high-sensitivity C-reactive protein (hsCRP), triglyceride, total lipoprotein cholesterol, high density lipoprotein (HDL) cholesterol, low density lipoprotein (LDL) cholesterol**.** Blood pressure was determined from the average of three automated sphygmomanometer measures after a ten minute supine rest. A sphygmomanometer (A & D Instruments Ltd., Japan) approved by the British Hypertension Society was used by operators trained in use of the device. Height was measured using a portable stadiometer accurate to 0.1 cm and weight using electronic scales accurate to 0.1kg. Body mass index was calculated. Homeostatic model assessment (HOMA) was applied to quantify insulin resistance based on insulin and glucose measures. (e1) Serum concentration of LDL cholesterol was calculated using Friedewald formula). (e2)

Appendix e-3. Volumetric assessment of total brain, white and grey matter

Volumetric assessment of total brain, white and grey matter was undertaken in T1 weighted sequences, with the following parameters: repetition time 12 ms, echo time 5.65 ms, in plane resolution 1.0 x 1.0 mm, slice thickness 1.0 mm. Application of the SIENAX algorithm to brain scans included the following protocol. Brain and skull images from the single whole-head input data were extracted. Brain images were registered to the Montreal Neurological Institute 152 space. Images were adjusted for skull size using the skull images to determine the registration scaling. Tissue

segmentation with partial volume estimation was carried out to calculate volumes for total (cortex and subcortical nuclei) and cortical grey matter as well as white matter. The rationale for undertaking brain volume assessment using automated segmentation was to minimize the chance of observer bias and increase accuracy.

Appendix e-4. White matter lesion evaluation

All analysed FLAIR images had the following parameters: repetition time: 9000 ms, echo time: 80 ms, flip angle:1800, in plane resolution: 1.0 x 1.0 mm, slice thickness: 3.0 mm. Visual analysis was undertaken using the MRI image analysis software package (CVI42® version 5.1.1, Circle Cardiovascular Imaging Inc., Calgary, Canada). Voxel based analysis of WML number and volume was performed using the following protocol. BIANCA was applied to classify the image's voxels based on their intensity (on FLAIR images) and spatial features (MNI coordinates), comparing them to a set of pre-classified voxels (9 images manually segmented by trained image analysts - TS, AB). The output image represents a probability of >0.9 per voxel of being WML. Volumes of WML were calculated within predefined masks for the total brain and the temporal, frontal, parietal and occipital lobes. The rationale for undertaking WML volume assessment using voxel-based automated segmentation was to minimize the chance of observer bias and increase accuracy.

Appendix e-5. Diffusion tensor imaging

A diffusion sequence was applied twice, with a 180^0 rotation in phase encoding direction for the second sequence (repetition time 7300 ms, echo time 81 ms, in plane resolution 2.0 x 2.0 mm, slice thickness 2.0 mm). Diffusion weighted images for

the entire cohort were aligned using nonlinear registration and a mean white matter skeleton for the cohort identified using voxels with values of fractional anisotropy greater than 0.2, thus identifying the centre of all fibre bundles. For each individual the diffusion data was overlaid onto this skeleton, each skeleton voxel representing the greatest fractional anisotropy value of the nearest fibre bundle. Mean values for the whole white matter skeleton overlay were then calculated, as well as regional values using predefined masks to exclude all but the lobe of interest. Mean diffusivity, fractional anisotropy, radial diffusivity and axial diffusivity were calculated for the total brain and separately for the temporal, frontal, parietal and occipital lobe.

Appendix e-6 Additional details on the study population

The causes of preterm delivery in study participants without hypertension during pregnancy were spontaneous preterm labour (10 participants), fetal indications such as fetal distress or intrauterine growth restriction (7 participants), premature rupture of membranes (2 participants) and it was not known in the remainder of cases (10 participants). None of our subjects were diagnosed with posterior reversible encephalopathy syndrome.

Appendix e-7 Subgroup analysis of comparing early versus late onset preeclampsia

An additional subgroup analysis was undertaken to compare the effects of early versus late onset preeclampsia on temporal lobe white matter lesions and cortical volume between. Group differences between women with previous early (<34 weeks' gestation) and late (≥34 weeks' gestation) onset preeclampsia were analysed using Student’s t–test or Mann-Whitney U test, according to data distribution. These

additional analyses showed no between-group differences in either cortical grey matter volumes or temporal lobe WML volume (table e-4).

E-references

e1. Friedewald WT, Levy RI, Fredrickson DS. Estimation of the concentration of low-density lipoprotein cholesterol in plasma, without use of the preparative ultracentrifuge. Clin Chem 1972;18:499-502.

e2. Okita K, Iwahashi H, Kozawa J, et al. Homeostasis model assessment of insulin resistance for evaluating insulin sensitivity in patients with type 2 diabetes on insulin therapy. Endocr J 2013;60(3):283-290.
