# Supplementary material for: Long-term cerebral white and gray matter changes after preeclampsia
Source: Neurology. 2017 Mar 28;88(13):1256–64. doi: 10.1212/WNL.0000000000003765 (PMC5373775; doi:10.1212/WNL.0000000000003765)
Supplement: Data Supplement [file supp_WNL.0000000000003765_e-tables.pdf]

Supplemental data: tables

Table e-1. Subcortical brain volumes adjusted to skull size

	Women with a history of normotensive pregnancy (n=49)	Women with a history of preeclampsia (n=34)	p-value
Thalamus (ml)	15.7±1.0	15.5±1.0	0.24
Caudate (ml)	6.6±0.6	6.6±0.7	0.71
Putamen (ml)	9.0±0.8	8.9±0.6	0.70
Pallidus (ml)	3.4±0.2	3.3±0.2	0.20
Hippocampus (ml)	6.5±0.8	6.5±0.6	0.94
Amygdala (ml)	2.4±0.3	2.5±0.4	0.79
Accumbens (ml)	0.9±0.2	0.8±0.1	0.29
Brainstem (ml)	20.2±1.9	20.1±1.8	0.78

Legend to table e-1. Bold numbers indicate statistical significance.

Table e-2. White matter lesion count

	Women with a history of normotensive pregnancy (n=49)	Women with a history of preeclampsia (n=34)	p-value
**Temporal lobe: number of lesions**			
Visual analysis	0 (0-2)	0 (0-4)	0.15
Voxel-based analysis	0 (0-3)	1 (0-6)	0.12
**Frontal lobe: number of lesions**			
Visual analysis	2 (0-8)	3 (0-24)	0.12
Voxel-based analysis	5 (1-15)	5.5 (0-20)	0.12
**Parietal lobe: number of lesions**			
Visual analysis	2 (0-4)	0 (0-4)	0.16
Voxel-based analysis	0 (0-6)	0 (0-6)	0.53
**Occipital lobe: number of lesions**			
Visual analysis	0 (0-2)	0 (0-4)	0.25
Voxel-based analysis	2 (0-5)	1.5 (0-5)	0.61

Legend to table e-2. Bold numbers indicate statistical significance. Mean±standard deviation and median (range)

Table e-3. Global and regional analyses of white matter lesion volumes and microstructural integrity

	Women with a history of normotensive pregnancy (n=49)	Women with a history of preeclampsia (n=34)	p-value
**Total brain**			
Lesion volume (µl)	412.6±257.5	546.6±570.8	0.15
Fractional anisotropy	0.491±0.01	0.486±0.01	0.10
Mean diffusivity (*10^{-6} mm^2/sec)	700±14	705±16	0.10
Axial diffusivity (*10^{-6} mm^2/sec)	1110±17	1110±21	0.40
Radial diffusivity (*10^{-6} mm^2/sec)	496±14	503±16	0.06
**Temporal lobe**			
Lesion volume (µl)	10.9±2.1	23.3±.44	**0.04**
Fractional anisotropy (nm/sec)	0.464±0.01	0.457±0.01	**0.03**
Mean diffusivity (*10^{-6} mm^2/sec)	719±18	730±19	**0.02**
Axial diffusivity (*10^{-6} mm^2/sec)	1100±22	1110±24	0.14
Radial diffusivity (*10^{-6} mm^2/sec)	526±18	538±019	**0.008**
**Frontal lobe**			
Lesion volume (µl)	223.3±19.8	318.8±70.4	0.12
Fractional anisotropy	0.484±0.01	0.480±0.01	0.21
Mean diffusivity (*10^{-6} mm^2/sec)	689±17	694±17	0.20
Axial diffusivity (*10^{-6} mm^2/sec)	1080±21	109±24	0.83
Radial diffusivity (*10^{-6} mm^2/sec)	493±16	498±17	0.17
**Parietal lobe**			
Lesion volume (µl)	0.88±0.2	1.09±0.23	0.52
Fractional anisotropy	0.510±0.011	0.504±0.01	0.14
Mean diffusivity (*10^{-6} mm^2/sec)	701±15	707±19	0.10
Axial diffusivity (*10^{-6} mm^2/sec)	1130±18	1140±24	0.45
Radial diffusivity (*10^{-6} mm^2/sec)	485±16	493±19	**0.05**
**Occipital lobe**			
Lesion volume (µl)	92.0±14.9	103.2±2	0.40
Fractional anisotropy	0.46±0.02	0.45±0.02	**0.04**
Mean diffusivity (*10^{-6} mm^2/sec)	749±18	757±22	0.10
Axial diffusivity (*10^{-6} mm^2/sec)	1160±26	1170±29	0.69
Radial diffusivity (*10^{-6} mm^2/sec)	542±20	552±23	**0.04**

Legend to table e-3. Bold numbers indicate statistical significance.

Table e-4. Subgroup comparison of grey matter volumes and temporal white matter lesion volume in women with previous early versus late onset preeclampsia

	Women with a history of early onset preeclampsia (n=21)	Women with a history of late onset preeclampsia (n=13)	p-value
Total grey matter volume (ml)	661.0±37.2	666.1±40.1	0.72
Cortical grey matter volume (ml)	371.1±24.6	380.4±27.8	0.64
Temporal lesion volume (µl)	25.7±25.2	19.6±24.8	0.47

Legend to table e-4. Bold numbers indicate statistical significance.
