# Supplementary material for: Long-term cerebral white and gray matter changes after preeclampsia
Source: Neurology. 2017 Mar 28;88(13):1256–64. doi: 10.1212/WNL.0000000000003765 (PMC5373775; doi:10.1212/WNL.0000000000003765)
Supplement: Accompanying Editorial [file supp_WNL.0000000000003765_1216.pdf]

Persistent brain injury after preeclampsia

Marilyn J. Cipolla, PhD
José Biller, MD

Correspondence to
Dr. Cipolla:
Marilyn.Cipolla@uvm.edu

Neurology® 2017;88:1216–1217

Preeclampsia, a common hypertensive disorder of pregnancy, contributes substantially to maternal and fetal morbidity and mortality worldwide. While the cause of preeclampsia remains debated, it is clear that there are both placental and maternal causes of preeclampsia, making it a heterogeneous disease.[1] Abnormal trophoblast invasion of spiral arteries during placentation leads to placental ischemia and release of placental-derived soluble factors into the maternal circulation.[2] These circulating proinflammatory and antiangiogenic factors appear to produce maternal vascular inflammation and cause endothelial dysfunction that underlies hypertension and proteinuria associated with preeclampsia.[1,2] However, not all women with preeclampsia have placental dysfunction. In these cases, the physiologic burden of pregnancy, characterized by mild peripheral inflammation, is thought to unmask preexisting maternal vascular dysfunction.[1] The presence of both maternal and placental disease leads to the most severe and early-onset form of the disorder.[1] Because of this heterogeneity, preeclampsia has been subclassified by clinical severity determined by maternal and fetal characteristics. Preeclampsia is considered severe if it is complicated by fetal growth restriction, which is strongly associated with placental dysfunction and early-onset disease. Subclassification of preeclamptic disease relating to time of delivery has also facilitated understanding the cause and complications of preeclampsia. For example, early-onset preeclampsia is a severe form of the condition involving maternal and placental disease in which delivery occurs ≤34 weeks of gestation, whereas late-onset preeclampsia is typically a more mild form of disease in which delivery occurs >34 weeks of gestation.[1]

It is becoming increasingly clear that women with a history of preeclampsia have excessive cardiovascular disease (CVD) risk later in life, especially those who have had severe early-onset preeclampsia. In a large retrospective cohort study, women with prior early-onset preeclampsia had a 9-fold increased risk of death from CVD.[3] Prior preeclampsia also increases the risk of future cerebrovascular disease. Women with a history of preeclampsia or eclampsia have a 2- to 4-fold increased risk of cerebrovascular disease and stroke later in life.[4,5] The increased risk of cerebrovascular disease in women with prior preeclampsia is also associated with subjective cognitive complaints and increased white matter lesion burden on MRI.[6,7] Whether the increased risk of cerebrovascular disease in these women is due to increased CVD risk factors that present with preeclampsia, or to structural and functional alterations to the cerebrovasculature in response to preeclampsia, remains unknown. In this issue of *Neurology*®, Siepmann et al.[8] attempted to address this question. This group used state-of-the-art MRI to assess regional and microstructural changes in white and gray matter in women with a history of preeclampsia, 5–15 years after the index pregnancy, compared to women with uncomplicated pregnancies. The authors used T1-weighted MRI to assess total brain and white and gray matter volume, T2-weighted MRI with fluid-attenuated inversion recovery to assess cerebral white matter lesion incidence and volume, and diffusion tensor imaging to investigate the microstructural integrity of white matter in women with former preeclampsia compared to women who had uncomplicated pregnancies. Importantly, these authors found that women who had prior preeclampsia had significantly reduced total gray matter volume compared to healthy controls. In addition, all women, regardless of preeclampsia, had frontal lobe white matter lesions. However, women who had preeclampsia had significantly increased temporal lobe white matter lesion volume with a loss of microstructural integrity (detected by changes in radial diffusivity) compared to women with healthy pregnancies. The increased temporal radial diffusivity in white matter in women with a history of preeclampsia correlated positively with time since index pregnancy but not with a higher CVD risk profile. White matter microstructural impairment did not associate with time from index pregnancy in women who had normal pregnancies. Thus, women with prior preeclampsia had white matter damage that appeared to increase over time, consistent with a continued susceptibility to injury that persists after

See page 1256

From the Departments of Neurological Sciences, Obstetrics, Gynecology & Reproductive Sciences and Pharmacology (M.J.C.), University of Vermont Larner College of Medicine, Burlington; and Department of Neurology (J.B.), Loyola University Chicago, Maywood, IL.

Go to Neurology.org for full disclosures. Funding information and disclosures deemed relevant by the authors, if any, are provided at the end of the editorial.

© 2017 American Academy of Neurology
© 2017 American Academy of Neurology. Unauthorized reproduction of this article is prohibited.

pregnancy. Longitudinal measurements will be needed to validate this conclusion, but the findings are consistent with previous studies showing greater white matter lesion burden and subjective cognitive impairment in women with prior preeclampsia compared to healthy pregnancies.[6,7] Another limitation of the study is that cognitive and autonomic function was not assessed, which may help to better elucidate mechanisms and consequences of cerebrovascular risk and early cognitive decline.

This study, together with others demonstrating increased CVD risk in women with a history of preeclampsia, suggests that more aggressive interventions may be useful to prevent cumulative brain damage. In fact, the American Heart Association recommends that a history of preeclampsia be considered a major risk factor for coronary heart and cerebrovascular disease.[9] For neurologists, assessment of pregnancy history may also be useful for prediction and prevention of cerebrovascular disease, stroke, and early cognitive impairment. However, given that prediction and prevention of preeclampsia is not currently feasible, there is a need for healthy lifestyle education in women with a history of preeclampsia and their offspring, considering that children from formerly preeclamptic women are also at increased risk of developing CVD and high blood pressure later in life.[10]

STUDY FUNDING

No targeted funding reported.

DISCLOSURE

The authors report no disclosures relevant to the manuscript. Go to Neurology.org for full disclosures.

REFERENCES

1. Staff AC, Benton SJ, von Dadelszen P, et al. Redefining preeclampsia using placenta-derived biomarkers. Hypertension 2013;61:932–942.
2. Roberts JM, Taylor RN, Musci TJ, Rodgers GM, Hubel CA, McLaughlin MK. Preeclampsia: an endothelial cell disorder. Am J Obstet Gynecol 1989;161:1200–1204.
3. Mongraw-Chaffin ML, Cirrillo PM, Cohn BA. Preeclampsia and cardiovascular disease death: prospective evidence from the child health and development studies cohort. Hypertension 2010;56:166–171.
4. Brown MC, Best KE, Pearce MS, Waugh J, Robson SC, Bell R. Cardiovascular disease risk in women with pre-eclampsia: systematic review and meta-analysis. Eur J Epidemiol 2013;28:1–19.
5. Wilson BJ, Watson MS, Prescott GJ, et al. Hypertensive diseases of pregnancy and risk of hypertension and stroke in later life: results from cohort study. BMJ 2003;326:845.
6. Postma IR, Bouma A, de Groot JC, Aukes AM, Aarnoudse JG, Zeeman GG. Cerebral white matter lesions, subjective cognitive failures, and objective neurocognitive functioning: a follow-up study in women after hypertensive disorders of pregnancy. J Clin Exp Neuropsychol 2016;38: 585–598.
7. Wiegman MJ, Zeeman GG, Aukes AM, et al. Regional distribution of cerebral white matter lesions years after preeclampsia and eclampsia. Obstet Gynecol 2014;123:790–795.
8. Siepmann T, Boardman H, Bilderbeck A, et al. Long-term cerebral white and gray matter changes after preeclampsia. Neurology 2017;88:1256–1264.
9. Mosca L, Benjamin EJ, Berra K, et al. Effectiveness-based guidelines for the prevention of cardiovascular disease in women—2011 update: a guideline from the American Heart Association. Circulation 2011;123:1243–1262.
10. Davis EF, Lewandowski AJ, Aye C, et al. Clinical cardiovascular risk during young adulthood in offspring of hypertensive pregnancies: insights from a 20-year prospective follow-up birth cohort. BMJ Open 2015;5:e008136.

© 2017 American Academy of Neurology. Unauthorized reproduction of this article is prohibited.
